# The Hong Kong infection control nurses’ association (HKICNA) – small establishment big impact

**DOI:** 10.1186/1753-6561-5-S6-P1

**Published:** 2011-06-29

**Authors:** CHS Lam, PTY Ching, WH Seto, W Jarvis, D Pittet

**Affiliations:** 1 Hospital Authority, Hong Kong, Hong Kong, China; 2 Jason and Jarvis Associates, South Carolina, USA; 3 Infection Control Program, University of Geneva Hospitals and Faculty of Medicine, Geneva, Switzerland; 4 WHO Collaborating Centre on Patient Safety, University of Geneva Hospitals, Geneva, Switzerland

## Introduction / objectives

In Hong Kong, the importance of infection prevention and control is recognized indubitably after an outbreak of MRSA with 6 neonatal deaths in 1985. The same year 24 infection control nurses were deployed full-time and formal training course was initiated. In 1989, HKICNA was inaugurated with 44 members and advisers. The followings are the milestones on how HKICNA, developed from a very small establishment, to have significant impacts on healthcare professionals in Hong Kong.

## Methods

The first 10 years of establishment, infection control awareness continued to grow. Initially the association focused on local meetings to promote and enhance knowledge in infection control. From 1999 HKICNA had ambitions to expand academically to start training course in 1999, publish biannual HKICNA newsletter in 2002.

2003 was a defining moment for infection control in Hong Kong because of the SARS outbreak. Training was in great demand. In 2003-2004, 10 classes with 3212 nurses and doctors trained from local, Macau and China. In that year alone, members grew to 1400 and HKICNA gained enough seed money to organize International Conference as a platform for exchange of scientific information and experience.

Since conception in 1989, we have flourished to a reputable academic nursing association in Hong Kong. Now HKICNA is providing the following activities: website, 6-monthly newsletters, Biannual International Conference, annual research grant award, annual conference sponsorships, training course, hand hygiene promotion in the community.

## Conclusion

Looking back, it is amazing that how a small establishment of a nursing association can instigate such impressive impact professionally.

## Disclosure of interest

None declared.

